# Supplementary material for: Measuring elimination of gambiense human African trypanosomiasis: a comparison of deceptively different metrics
Source: Parasit Vectors. 2025 Dec 1;19:8. doi: 10.1186/s13071-025-07131-0 (PMC12771873; doi:10.1186/s13071-025-07131-0)
Supplement: Supplementary file 1 — Additional file 1: French language version [file 13071_2025_7131_MOESM1_ESM.pdf]

Mesurer l'élimination de la trypanosomiase humaine Africaine *gambiense* : Une comparaison de mesures trompeusement différentes

Samuel A. Sutherland[1,2*], Jason J. Madan[1,2] and Kat S. Rock[1,3]

[1*]The Zeeman Institute for Systems Biology & Infectious Disease Epidemiology Research, University of Warwick, University Road, Coventry, CV4 7AL, United Kingdom.
[2]Centre for Health Economics at Warwick, Warwick Medical School, University of Warwick, University Road, Coventry, CV4 7AL, United Kingdom.
[3]Warwick Mathematics Institute, University of Warwick, University Road, Coventry, CV4 7AL, United Kingdom.

*Corresponding author(s). E-mail(s): Samuel.A.Sutherland@warwick.ac.uk;
Contributing authors: J.J.Madan@warwick.ac.uk; K.S.Rock@warwick.ac.uk;

Résumé

La modélisation est un outil efficace et très répandu pour prédire la trajectoire future des maladies infectieuses. L'une de ses forces réside dans sa capacité à mesurer et à prédire des paramètres qui ne sont pas observables dans la vie réelle, tels que la prévalence réelle ou le moment où les infections se produisent. Les objectifs politiques, par contre, doivent nécessairement être basés sur des paramètres observables afin d'être vérifiables. L'Organisation Mondiale de la Santé (OMS) a fixé comme objectif l'élimination de la transmission de la trypanosomiase humaine africaine *gambiense* (THAg) d'ici 2030. Afin de vérifier cet objectif, le groupe consultatif technique de l'OMS sur l'élimination de la THA (HAT-e-TAG) a récemment défini l'indicateur national pour l'EdT comme étant l'absence, pendant cinq années, de nouveaux cas d'infection locale signalés, accompagnée d'un niveau de surveillance suffisant. Bien que cet indicateur soit une mesure utile et concrète qui peut être clairement définie, il ne mesure pas directement la transmission ou la prévalence réelle de la maladie. Dans certains

cas, la date du dernier événement de transmission ou le moment où l'infection n'est plus présente dans un pays peut être très différent du moment où le pays atteint l'indicateur de l'OMS pour l'élimination de la transmission. Dans cet article, nous discutons de la différence entre ces différentes mesures et montrons, à l'aide de quelques exemples, à quel point elles peuvent diverger. En outre, des articles de modélisation ont été publiés jusqu'à présent en utilisant diverses approximations de l'EdT, ce qui peut prêter à confusion. Dans cet article, nous soulignons les malentendus potentiels qui pourraient se produire en raison de différences apparemment insignifiantes dans la définition de l'élimination et de ses indicateurs, en particulier dans le contexte d'une maladie qui progresse aussi lentement que la THAg. Nous concluons que bien que les modèles prédisant des paramètres observables et non observables soient utiles, les modélisateurs doivent s'assurer que les définitions sont claires lorsqu'ils communiquent les résultats, et les décideurs doivent s'assurer qu'ils savent quelles définitions sont utilisées lorsqu'ils tirent des conclusions à partir des résultats des modèles. Bien que cet article se concentre spécifiquement sur trois indicateurs de l'EdT pour la THAg, les points généraux soulevés ici pourraient s'appliquer à d'autres infections approchant de la phase finale, telles que l'onchocercose, ou même au-delà de la santé publique.

Keywords: élimination, trypanosomiasise humaine Africaine (THA), maladie de sommeil, modélisation, maladies tropicales negligés (MTN), surveillance des maladies, terminologie, Organisation Mondiale de la Santé (OMS)

Definir l'élimination de la THAg

La tryanosomiase humaine Africaine (THA) *gambiense* (THA) est une infection parasitaire à transmission vectorielle endémique dans les régions d'Afrique de l'Ouest et d'Afrique centrale. Si elle n'est pas traitée pendant plusieurs années, l'infection entraîne généralement, mais pas toujours, la mort [1]. Il n'existe pas de vaccin pour protéger contre l'infection et les efforts de lutte reposent généralement sur la détection et le traitement, parfois accompagnés d'une lutte antivectorielle afin de réduire les possibilités de transmission par les tsé-tsé vecteurs. La THA*g* a été ciblée par l'Organisation Mondiale de la Santé (OMS) pour élimination comme problème de santè publique (EPSP) d'ici 2020 [2] — un objectif qui a manqué de peu son année cible [3] mais qui a été atteint juste deux ans plus tard [4] — et a également été marquée pour l'élimination de la transmission (EdT) à l'horizon 2030 dans le cadre de la *feuille de route pour les maladies tropicales negligées (MTN) 2021–2030.* [5]. Alors que l'indicateur OMS d'élimination en tant que problème de santé publique (EPSP-OMS) est entièrement défini par des rapports de cas mesurables, l'EdT est malheureusement plus difficile à mesurer. Il n'est pas possible d'observer directement les événements de transmission à l'origine des nouvelles infections, il faut donc un indicateur indirect pour pouvoir mesurer les progrès accomplis dans la réalisation de cet objectif. En particulier, la THA*g* a une durée d'infection très longue (souvent plusieurs années, parfois plus d'une décennie [6]), ce qui signifie que les cas sont souvent découverts considérablement après que le patient a été initialement infecté.

En novembre 2023, le Groupe consultatif technique sur l'élimination de la trypanosomiase humaine africaine (HAT-e-TAG) de l'OMS a publié son nouvel ensemble de critères qui seront utilisés pour évaluer si les pays ont atteint le seuil de vérification de l'EdT [7]. Les indicateurs de l'EdT sont différents selon l'infection et dépendent de divers facteurs, notamment la durée de l'infection, les méthodes de diagnostic disponibles pour détecter l'agent pathogène et les stratégies de surveillance réalisables [5]. Par exemple, l'indicateur de l'EdT au niveau national pour l'onchocercose comprend la mesure de la prévalence chez le vecteur des simulies ainsi que l'échantillonnage chez les enfants – plus précisément, <0,1% de séropositivité Immunoglobulin G4 (IgG4) à l'antigène Ov-16 *Onchocerca volvulus* chez les enfants de moins de 10 ans, et <0.05% de positivité par dépistage en pool amplification en chaîne par polymérase (PCR) dans au moins 6 000 mouches sauvages capturées (têtes uniquement)[8]. Pour la THA*g*, l'indicateur défini par le HAT-e-TAG pour l'EdT dans un pays donné est une période de cinq ans au cours de laquelle aucun nouveau cas d'infection locale n'a été signalé dans le pays, sous réserve d'un nombre suffisant d'activités de recherche de cas, comme le précisent leurs lignes directrices complètes [7].

Les cas de la THAg sont généralement identifiés par un test de dépistage sérologique initial, soit le card agglutination test for trypanosomiasis (CATT), soit un teste diagnostique rapide (TDR), suivi d'une microscopie de confirmation pour visualiser le parasite dans le sang. Tous les traitements actuellement disponibles peuvent avoir des effets secondaires importants, par conséquent le traitement ne peut être administré qu'aux patients dont l'infection par le parasite est confirmée, ce qui signifie que l'administration massive de médicaments (AMM) n'est actuellement pas une option. Bien que le traitement ait été amélioré en 2020 pour la plupart des cas grâce au lancement du médicament oral fexinidazole, celui-ci n'est encore généralement indiqué que pour les cas confirmés [10], bien que certains pays traitent les cas fortement suspects sur la base de la sérologie. En outre, certains cas doivent encore être traités par voie intraveineuse à l'hôpital, en fonction de l'âge, du poids ou de la gravité de la maladie [10]. Plus récemment, un nouveau médicament connu sous le nom d'acoziborole a été développé [11] et des essais sont actuellement en cours pour évaluer sa sécurité d'utilisation dans les cas suspects [12, 13]. Malgré cela, il n'est pas prévu d'utiliser l'acoziborole chez les patients ne présentant pas de suspicion sérologique de la THA*g*.

Les cas sont généralement diagnostiqués par l'une des deux voies suivantes : le dépistage « actif » ou « passif ». Le dépistage actif consiste en un dépistage de masse des participants volontaires dans les zones à risque, soit par le CATT, soit par les TDR [14]. Le dépistage passif fait référence aux personnes infectées qui se présentent dans des établissements de santé fixes avec des symptômes spécifiques de la maladie de la THA*g*, et qui sont ensuite testées pour cette maladie [15]. Cela dépend à la fois de la disponibilité du TDR dans l'établissement où le patient se présente et de la sensibilisation du prestataire de soins aux symptômes de la THA*g*. Cela signifie que la détection est nécessairement très liée à l'intervention, contrairement à beaucoup d'autres MTN où AMM est l'intervention principale et où la surveillance est généralement un processus distinct.

Un article précédent décrivait certains des défis associés à la mesure de l'EdT pour la THA*g* en utilisant les rapports de cas [16], soulignant que le niveau des

**Indicateurs de l'OMS pour la THA*g***	
**Mesure**	**Description**
Indicateur de l'OMS de l'élimination en tant que problème de santé publique (EPSP-OMS) au niveau global	(1) Moin que 2 000 cas signalés par an; et (2) une réduction de 90% de la zone à risque déclarant $\geq$ 1 case/10 000 personnes/an (moyenne sur une période de 5 ans) d'ici 2016–2020 par rapport à la base de référence 2000–2004 [9].
Indicateur de l'OMS de l'élimination en tant que problème de santé publique (EPSP-OMS) au niveau du pays	$\leq$ 1 cas/10 000 personnes/an (moyenne sur une période de 5 ans), dans chaque district sanitaire du pays (sous réserve d'un contrôle et d'une surveillance fonctionnels)[9].
Indicateur de l'OMS de l'élimination de la transmission (EdT-OMS) au niveau du pays	Au moins cinq années consécutives sans aucun nouveau cas humain local dans un pays donné (avec preuve d'une surveillance appropriée). Cela exclut les cas dont on peut prouver qu'ils ne sont pas nouveaux ou les infections locales. [7].
**Stades réels d'élimination (non observables)**	
**Mesure**	**Description**
Dernier événement de transmission (DET) in a region	Il n'y a plus d'événements de transmission. L'infection peut subsister chez certains hôtes précédemment infectés et des cas peuvent être détectés pendant une longue période après que cet état a été atteint, mais il n'y a pas de transmission ultérieure. Il n'est pas possible d'observer directement si cet état a été atteint.
Aucune infection restante (AIR) dans une région	Plus aucun humain, vecteur ou autre espèce hôte potentielle infecté par THA*g* dans une région donnée. À moins d'une réimportation, THA*g* n'est plus présent dans cette région. Il n'est pas possible d'observer directement si cet objectif a été atteint.
**Approximations modélisées de l'élimination**	
**Mesure**	**Description**
Indicateur de l'OMS de l'élimination en tant que problème de santé publique modélisé (EPSP-OMS-modélisé) dans une région	$\leq$ 1 cas/10,000 personnes/an (moyenne sur une période de 5 ans), dans une région spécifique.
Indicateur de l'OMS de l'élimination de la transmission modélisé (EdT-OMS-modélisé) dans une région	Au moins 5 années consécutives sans nouveau cas humain dans une région spécifique.
Dernier événement de transmission modélisé (DET-modélisé) dans une région	Dernière année de la simulation au cours de laquelle un événement de transmission se produit. Aucun autre événement de transmission ne se produit après cette date.
Aucune infection restante (AIR-modélisé) dans une région	La première année où il n'y a plus d'infection dans le modèle dans une région spécifique.

Tableau 1 List de mesures de l'élimination de la THA*g* et leurs définitions.

activités actives et passives de recherche de cas et la présence ou l'absence de lutte antivectorielle pouvaient influencer la mesure de l'EdT ; cependant, au moment de la publication, l'indicateur officiel de l'OMS pour la THAg n'avait pas encore été publié.

Dans cet article, nous cherchons à mieux comprendre comment l'indicateur EdT-OMS (qui peut être directement mesuré) est lié au DET et AIR (comme définis dans le Tableau 1) qui ne peuvent pas être directement observés. Nous évaluons cela à l'aide d'un cadre de modélisation, en comparant le point auquel l'indicateur de l'OMS est atteint, le point auquel la transmission cesse réellement et le point auquel aucune infection ne subsiste. Nous visons également à clarifier la différence entre l'indicateur EdT-OMS et la définition de l'EdT utilisée dans les articles de modélisation précédents de notre groupe et d'autres auteurs.

Dans cet article, nous nous concentrerons spécifiquement sur la THA*g*, mais les points généraux soulevés ici s'appliquent à toutes les maladies infectieuses ciblées pour l'EdT ou l'éradication.

Quelques exemples illustratifs

La Figure 1 montre deux exemples d'histoires d'infections potentielles pour un endroit hypothétique approchant de l'élimination de la THA*g*. Dans l'*Exemple 1*, nous voyons la progression de la transmission et de l'infection dans une région où le dépistage actif et passif sont efficaces et où le niveau de transmission est faible, potentiellement en raison de la lutte antivectorielle. Ici, nous constatons que la dernière transmission a lieu en 2025, mais que la personne infectée n'est détectée que deux ans plus tard, au début de l'année 2027. En appliquant la règle de l'indicateur OMS pour d'EdT, cela signifierait que la première des cinq années d'absence de notification de cas ne pourrait pas commencer avant 2028, pour arriver finalement aux cinq années d'absence de cas pour OMS-EoT en 2033, cinq ans après qu'il n'y ait plus d'infection latente, et sept ans après le dernier événement de transmission.

Dans l'*Exemple 2*, nous voyons un exemple de la même région avec des interventions plus faibles, consistant en un dépistage moins efficace, où la transmission se poursuit plus de cinq ans après le dernier cas signalé. Pour cette raison, le DET se produit en fait après cinq ans d'absence de cas déclarés, et après le DET, il reste encore une personne infectée pendant quelques années. L'indicateur OMS étant basé sur la déclaration des cas, il dépend fortement du niveau de surveillance. Cela souligne l'importance de la composante surveillance de la définition de l'EdT-OMS, car sans un suivi suffisant, il est impossible de faire la différence entre l'absence d'infection et le manque de détection de l'infection qui subsiste.

Modélisation de l'élimination et de ses indicateurs

La modélisation mathématique a été utilisée pour répondre à une série de questions quantitatives sur la transmission de la THAg, y compris l'évaluation du succès des interventions historiques sur la transmission et la recherche de cas, l'évaluation de l'impact attendu d'une série de stratégies futures sur la dynamique de l'infection et l'analyse des coûts et du coût-efficacité des stratégies d'intervention. Bien que des mesures telles que DET et AIR ne soient pas directement mesurables dans le monde

réel, nous pouvons utiliser des simulations de modèles pour calculer ces mesures à partir des résultats du modèle. De même, nous pouvons estimer le temps nécessaire pour atteindre EdT-OMS en utilisant les rapports de cas simulés.

Notre équipe de modélisation a précédemment développé et publié un modèle de transmission THAg que nous avons ajusté aux données de cas et utilisé dans une variété de contextes pour simuler la dynamique de l'infection [17–22]. Notre modèle est un modèle de transmission compartimentale avec une modélisation explicite de la dynamique des humains et des tsé-tsé, ainsi que de la stratification du risque chez les humains et de la possibilité d'avoir des hôtes asymptomatiques humains ou animaux non-humains. Dans cet article, nous utilisons une version stochastique de notre modèle qui incorpore des événements aléatoires et produit des nombres entiers d'infections et de cas. Un modèle stochastique a été choisi ici car les nombres entiers de personnes dans chaque état d'infection et d'événements permettent la lecture directe du DET-Modélisé et AIR-Modélisé lorsque les valeurs appropriées atteignent zéro dans le modèle. Des travaux antérieurs ont comparé l'efficacité de différentes interventions dans divers contextes en utilisant des variantes de ce modèle. Les auteurs de cet article ont précédemment utilisé un modèle déterministe, qui est plus simple à simuler et reflète la dynamique moyenne, mais ce type de modèle ne peut pas atteindre exactement zéro infection et a donc besoin d'un seuil de substitution pour déterminer le moment où l'élimination est atteinte. Cette technique donne généralement des résultats similaires pour l'année prévue de l'EdT, mais un modèle stochastique comme celui utilisé dans cet article produit généralement un éventail plus large de prévisions pour l'EdT, c'est-à-dire plus d'incertitude [23].

Parmi les résultats du modèle figurent des estimations de la probabilité d'atteindre différents indicateurs de l'EdT pour chaque année donnée. Pour le présent travail, nous avons utilisé le modèle pour produire chacune des mesures régionales détaillées dans le tableau 1, en utilisant des projections basées sur une région exemplaire, qui reflète encore la transmission THAg « typique ». Par souci de simplicité, nous n'avons pas inclus la possibilité d'infections humaines asymptomatiques dans ces projections, même si cela ne changerait rien aux points d'illustration que nous allons mettre en évidence. Nous avons inclus la possibilité d'infections animales dans un modèle d'ensemble comme dans [24].

Afin d'évaluer et de comparer les différents mesures de l'élimination, nous avons effectué des simulations dans le cadre de différents scénarios d'intervention et enregistré le moment où chaque mesure de l'élimination a été atteinte dans chaque réalisation. Nous avons considéré huit lieux hypothétiques différents avec une taille de population fixe de 100 000 personnes à partir de l'équilibre endémique de l'année 0, et avec des paramètres basés sur nos ajustements de modèles existants pour l'une des huit zones de santé différentes de la République Démocratique du Congo (RDC) afin de capturer une gamme de dynamiques d'infection plausibles et de confirmer que nos résultats seraient qualitativement similaires même si des paramètres tels que le ratio tsé-tsé-humain ou le délai de détection passive variaient. Nous avons réalisé ces simulations pendant deux ans avec un niveau faible de dépistage passif uniquement, puis nous avons simulé de l'année 2 à l'année 200 avec trois stratégies différentes, couvrant différents niveaux de dépistage passif, de dépistage actif et de lutte antivectorielle. La

première prévoit un dépistage actif de 10 % de la population chaque année, une lutte antivectorielle avec une réduction de 80 % après un an et un dépistage passif efficace (comparable à la couverture actuelle, avec un délai moyen entre l'infection et la détection de 2,7 ans en l'absence de dépistage actif selon le paramétrage utilisé dans la Figure 2). La deuxième est identique, mais sans lutte antivectorielle, et la troisième a une surveillance pire avec seulement 2 % de couverture de dépistage actif par an et un faible dépistage passif (comparable à la couverture estimée pour le début des années 2000, le temps moyen entre l'infection et la détection de 3,3 ans s'il n'est pas trouvé dans le dépistage actif sous la paramétrisation utilisée dans la Figure 2). Comme nous n'avons pas utilisé directement les tendances des cas ou l'historique des interventions correspondant à des lieux réels, cette analyse de modélisation illustrative ne reflète pas les progrès ou les prévisions pour des zones de santé spécifiques en RDC.

La Figure 2 montre les résultats en un lieu pour les stratégies qui vont de l'intervention lourde à l'intervention minimale. Nous pouvons voir sur la figure qu'à travers les différentes stratégies, la métrique EdT-OMS-Modélisé est conservatrice par rapport à DET-Modélisé et AIR-Modélisé. La taille exacte du retard dépend des facteurs d'infection spécifiques au lieu et du niveau d'intervention, mais dans la majorité de nos simulations, l'ordre est le même. Tout d'abord, DET-Modélisé est atteint, puis AIR-Modélisé, et enfin EdT-OMS-Modélisé. En effet, AIR doit nécessairement venir après EDT et, dans la plupart des scénarios où la surveillance est plus efficace, l'infection finale a plus de probabilité d'être déclarée comme un cas que de mourir sans avoir été déclarée et sans avoir provoqué d'autre infection. Nous constatons également que dans tous les scénarios avec un bon niveau de dépistage passif, il est presque certain que DET-Modélisé se produit avant EdT-OMS-Modélisé, avec un délai moyen d'environ 7 ans sans lutte antivectorielle, et de 10 ans avec la lutte antivectorielle. Les résultats pour les sept autres contextes sont présentés dans nos informations complémentaires et montrent un résultat qualitativement similaire. Si la surveillance passive est faible, il existe également une probabilité modérée qu'il n'y ait pas de notification de cas pendant 5 ans avant l'indicateur DET, c'est pourquoi nous considérons qu'une surveillance efficace est une stipulation importante de l'indicateur EdT-OMS.

Barrières linguistiques interdisciplinaires et interprétation pour la politique

Notre modélisation a montré que ces différentes mesures, bien qu'elles soient toutes nominalement des mesures d'élimination d'un certain type, peuvent être atteintes à des moments très différents. Les décideurs doivent être très attentifs à la manière dont les modélisateurs simulent l'EdT dans leurs résultats s'ils veulent les utiliser — utilisent-ils la définition de l'indicateur EdT-OMS pour l'EdT ou modélisent-ils DET ou AIR ? Par exemple, une étude de modélisation pour l'onchocercose [25] précise qu'elle utilise une définition plus proche de ce qui est appelé dans le présent rapport AIR, à savoir une « absence de parasites chez les humains et les mouches 50 ans après l'arrêt du traitement ». Les études, y compris les prévisions d'élimination de la THA*g* par le groupe « HAT Modelling and Economic Predictions for Policy (modélisation et prévisions économiques pour la politique relative à la trypanosomiase humaine africaine) (HAT MEPP) », auquel les auteurs de cet article sont affiliés, ont

précédemment défini l'EdT comme la première année après « le dernier événement simulé de transmission à l'homme », ce qui équivaut ici à un an après DET-Modélisé. (des exemples récents incluent [19–22]), ce qui est susceptible de ne pas correspondre à la date à laquelle l'indicateur EdT-OMS sera atteint. À l'avenir, le groupe HAT MEPP adoptera la présentation des indicateurs DET-Modélisé et EdT-OMS-Modélisé afin d'établir une distinction plus claire entre les prévisions relatives aux événements de transmission et la déclaration des cas.

D'une manière générale, la communauté MTN doit faire attention à ne pas assimiler les mêmes mots pour signifier des choses légèrement différentes—par exemple, « élimination » (sans qualificatif) pourrait être EPSP ou EdT—bien que ces dernières années cela se soit amélioré avec l'OMS qui a pris l'initiative de produire des définitions plus claires et de mettre à jour la documentation. Les modélisateurs devraient également s'efforcer de combler le fossé linguistique entre la description des résultats de la modélisation et la manière dont les cliniciens ou les décideurs politiques comprennent la terminologie ; la récente publication de l'indicateur EdT-OMS pour la THA*g* [7] a rendu la distinction entre ces différentes mesures beaucoup plus facile à expliquer. Nous recommandons aux modélisateurs qui produisent des projections d'élimination d'être très clairs sur les définitions qu'ils utilisent, voire de publier des projections utilisant plusieurs indicateurs. En outre, les modélisateurs peuvent également parler de l'EdT au niveau local ou régional, alors que les indicateurs de l'OMS ne sont actuellement définis qu'au niveau national, et il convient donc de prêter attention à l'échelle spatiale considérée.

Si toutes ces mesures ont le mérite de mesurer les progrès accomplis, il est essentiel que les décideurs, les modélisateurs et les autres parties prenantes sachent clairement quelle.s mesure.s ils utilisent et que de fausses équivalences ne soient pas établies entre deux mesures qui peuvent sembler similaires au départ, mais qui ne sont en fait pas les mêmes.

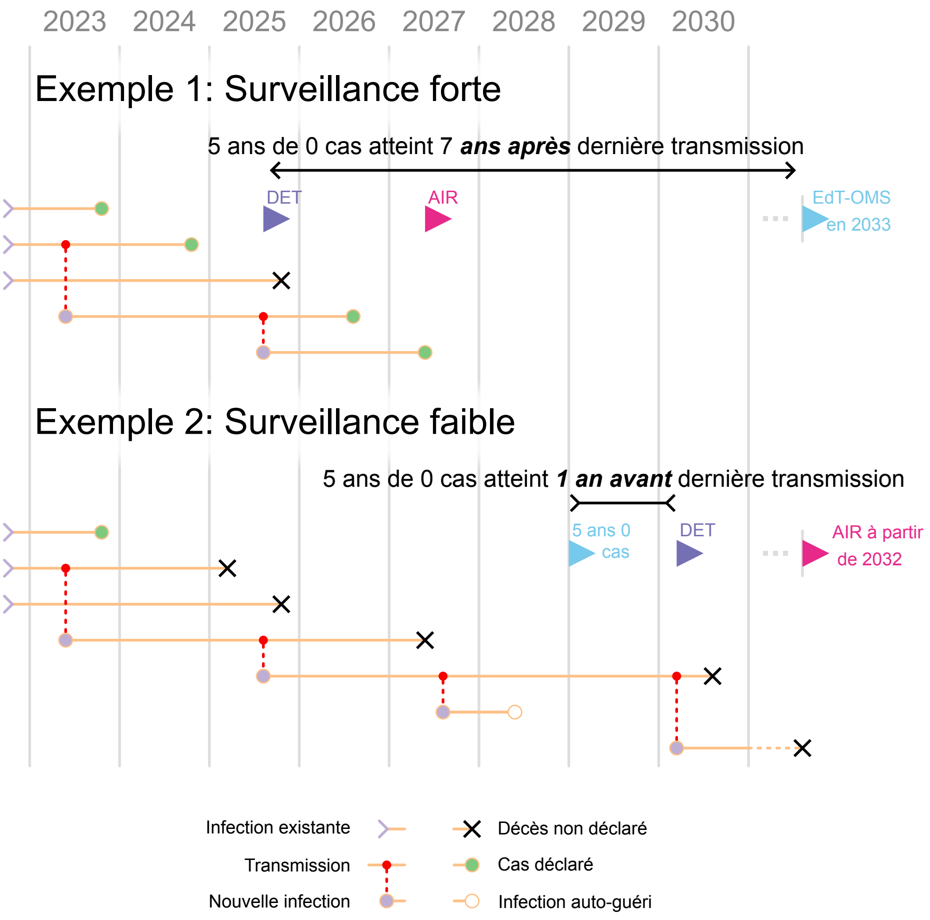

Fig. 1 Deux illustrations des voies possibles vers l'élimination au niveau du pays en partant d'une très faible prévalence de l'infection par la THA*g*. Tous les exemples commencent avec trois infections existantes en 2022 et les lignes horizontales oranges montrent les individus qui continuent d'être infectieux. Nous supposons que l'infection se termine soit par une détection et un traitement ultérieur (indiqués par des cercles verts), soit par un décès non signalé (indiqué par des croix noires), soit par une infection potentielle qui se guérit d'elle-même et qui n'est pas signalée (indiquée par un cercle non rempli). Les drapeaux de couleur indiquent la réussite des trois mesures d'élimination : cinq ans sans cas/EdT-OMS (bleu), DET (violet) et AIR (rose). Notez que dans le deuxième exemple, nous parlons de « 5 ans 0 cas » au lieu de EdT-OMS parce qu'il est peu probable qu'un tel contexte réponde aux critères de la mesure de l'OMS de l'EdT en raison d'une surveillance insuffisante.

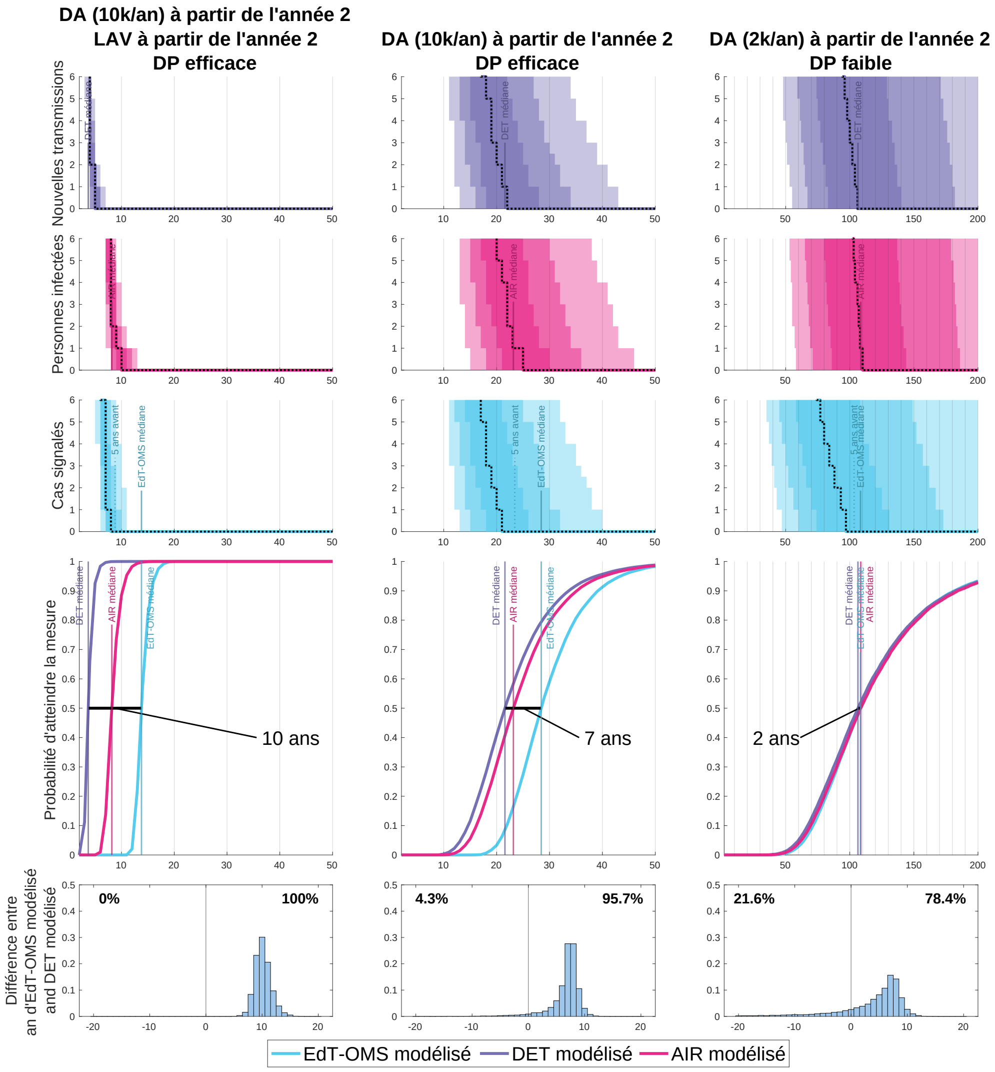

Fig. 2 Simulations du modèle montrant divers résultats et leur lien avec trois mesures d'élimination différentes. Les trois premiers graphiques de chaque colonne indiquent le nombre de nouvelles infections (violet) et le nombre de cas chaque année (bleu), ainsi que le nombre d'infections restantes à un moment donné (rose). Les lignes continues indiquent la valeur médiane du modèle, les intervalles de prédiction de 50 %, 80 % et 95 % étant indiqués par les régions ombrées. La quatrième figure présente des estimations de la probabilité de l'EdT-OMS-Modélisé, de la DET-Modélisé et d'AIR-Modélisé par année, calculées depuis la proportion des 20 000 simulations stochastiques du modèle qui ont atteint la mesure en question. La figure du bas présente un histogramme du retard entre DET-Modélisé et EdT-OMS-Modélisé. Les pourcentages indiquent la proportion de réalisations dans lesquelles l'événement de transmission final se produit respectivement après ou avant les cinq années d'absence de cas requises pour EdT-OMS. Les paramètres utilisés pour générer les simulations utilisées dans cette figure étaient basés sur le paramétrage de notre modèle pour la zone de santé de Bagata dans la coordination Bandundu Nord en RDC, bien que, comme nous n'avons pas directement utilisé les tendances des cas ou l'historique des interventions ici, cette analyse ne reflète pas les progrès ou les prédictions pour la zone de santé réelle.

Liste des abréviations

AIR	aucune infection restante.
AMM	administration massive de médicaments.
CATT	card agglutination test for trypanosomiasis.
DET	dernier évenement de la transmission.
EdT	élimination de la transmission.
EPSP	élimination comme problème de santè publique.
HAT MEPP	HAT Modelling and Economic Predictions for Policy (modélisation et prévisions économiques pour la politique relative à la trypanosomiase humaine africaine).
HAT-e-TAG	Groupe consultatif technique sur l'élimination de la trypanosomiase humaine africaine.
IgG4	Immunoglobulin G4.
MTN	maladie tropicale negligée.
OMS	Organisation Mondiale de la Santé.
PCR	amplification en chaîne par polymérase.
RDC	République Démocratique du Congo.
TDR	teste diagnostique rapide.
THA	tryanosomiase humaine Africaine.
THA*g*	THA *gambiense*.

Déclarations

Approbation éthique et consentement à participer

Cette étude de simulation n'a pas utilisé directement de données de cas humains et s'est appuyée sur des paramétrisations de modèles déjà publiées, issues d'autres articles de modélisation accessibles au public. Aucune nouvelle collecte de données n'a eu lieu dans le cadre de cette étude de modélisation.

Consentement à la publication

Non applicable

Disponibilité des données et du matériel

Le code source complet, les données utilisées et les résultats complets sont disponibles à l'adresse http://doi.org/10.17605/OSF.IO/N4DKZ

Intérêts concurrents

Les auteurs ont déclaré qu'ils n'avaient aucun conflit d'intérêts

Financement

Ce travail a été soutenu par la Fondation Gates (www.gatesfoundation.org) dans le cadre du projet de modélisation et de prévisions économiques pour la politique relative à la trypanosomiase humaine africaine (HAT MEPP) [INV-005121] (SAS, JM et KSR). Les financeurs de l'étude n'ont joué aucun rôle dans la conception, la collecte, l'analyse et l'interprétation des données, ni dans la rédaction du rapport.

Contributions des auteurs

SS et KR ont rédigé l'article. SS a réalisé des simulations et dessiné des figures. Tous les auteurs ont lu et approuvé le manuscrit final.

Remerciements

Merci à toute l'équipe du programme HAT MEPP et à ses collaborateurs pour les discussions continues qui ont inspiré la rédaction de cet article et pour le développement du code utilisé pour générer les résultats présentés ici. Les auteurs tiennent à remercier le Dr Erick Mwamba Miaka et l'équipe de l'Atlas de la THA de l'OMS pour l'accès aux données historiques de la RDC, qui ont permis la paramétrisation originale du modèle dans Antillon et al.[24]. Les auteurs tiennent également à remercier les Drs Paul Bessell, Vincent Jamonneau, Veerle Lejon, Elena Nicco et Iñaki Tirados pour leur contribution à la terminologie utilisée dans cet article.

Dans un souci d'accès libre, les auteurs ont appliqué une licence Creative Commons Attribution (CC-BY) à toute version de manuscrit acceptée par l'auteur issue de cette soumission.

Références

[1] Jamonneau V, Ilboudo H, Kaboré J, Kaba D, Koffi M, Solano P, et al. Untreated human infections by *Trypanosoma brucei gambiense* are not 100% fatal. PLoS Neglected Tropical Diseases. 2012;6(6):e1691.

[2] World Health Organization. Sustaining the drive to overcome the global impact of neglected tropical diseases: second WHO report on neglected diseases. WHO/HTM/NTD/2013.1. World Health Organization; 2013.

[3] Franco JR, Cecchi G, Paone M, Diarra A, Grout L, Kadima Ebeja A, et al. The elimination of human African trypanosomiasis: Achievements in relation

to WHO road map targets for 2020. PLoS Neglected Tropical Diseases. 2022;16(1):e0010047.

[4] Franco JR, Priotto G, Paone M, Cecchi G, Ebeja AK, Simarro PP, et al. The elimination of human African trypanosomiasis: Monitoring progress towards the 2021–2030 WHO road map targets. PLoS Neglected Tropical Diseases. 2024 04;18(4):1–22. https://doi.org/10.1371/journal.pntd.0012111.

[5] World Health Organization. Ending the neglect to attain the Sustainable Development Goals: a road map for neglected tropical diseases 2021–2030. World Health Organization; 2020.

[6] Sudarshi D, Lawrence S, Pickrell WO, Eligar V, Walters R, Quaderi S, et al. Human African trypanosomiasis presenting at least 29 years after infection—what can this teach us about the pathogenesis and control of this neglected tropical disease? PLoS Neglected Tropical Diseases. 2014;8(12):e3349.

[7] World Health Organization and others. Criteria and procedures for the verification of elimination of transmission of *T. b. gambiense* to the human population in a given country. World Health Organization; 2023.

[8] Organization WH, et al. Guidelines for stopping mass drug administration and verifying elimination of human onchocerciasis: criteria and procedures. WHO/HTM/NTD/PCT/2016.1. World Health Organization; 2016.

[9] Franco JR, Cecchi G, Priotto G, Paone M, Diarra A, Grout L, et al. Monitoring the elimination of human African trypanosomiasis at continental and country level: Update to 2018. PLoS neglected tropical diseases. 2020;14(5):e0008261.

[10] World Health Organization. Guidelines for the treatment of human African trypanosomiasis. World Health Organization; 2024.

[11] Kumeso VKB, Kalonji WM, Rembry S, Mordt OV, Tete DN, Prêtre A, et al. Efficacy and safety of acoziborole in patients with human African trypanosomiasis caused by Trypanosoma brucei gambiense: a multicentre, open-label, single-arm, phase 2/3 trial. The Lancet Infectious Diseases. 2023;23(4):463–470.

[12] ClinicalTrialsgov.: An Intervention Study to Evaluate the Impact of Treating gHAT Seropositive Subjects With Acoziborole on Transmission of T.b. Gambiense, and Obtain Further Safety Data on Acoziborole in gHAT Seropositive Individuals. Drugs for Neglected Diseases. Available from: https://clinicaltrials.gov/study/NCT06356974.

[13] ClinicalTrialsgov.: Safety and Tolerability Study of Acoziborole in g-HAT Seropositive Non-parasitologically Confirmed Subjects: a Multicentre Randomised Double-blind Placebo-controlled Study. Drugs for Neglected Diseases. Available from: https://clinicaltrials.gov/study/NCT05256017.

[14] World Health Organization. Report of the fifth WHO stakeholders meeting on gambiense and rhodesiense human African trypanosomiasis elimination. World Health Organization; 2024.

[15] World Health Organization and others. Control and surveillance of human African trypanosomiasis: report of a WHO expert committee. World Health Organization; 2013.

[16] NTD Modelling Consortium Discussion Group on *G*ambiense Human African Trypanosomiasis. Insights from quantitative and mathematical modelling on the proposed 2030 goal for *gambiense* human African trypanosomiasis (gHAT) [version 2; peer review: 2 approved]. Gates Open Research. 2020;3:1553.

[17] Rock KS, Torr SJ, Lumbala C, Keeling MJ. Quantitative evaluation of the strategy to eliminate human African trypanosomiasis in the Democratic Republic of Congo. Parasites & Vectors. 2015;8:1–13.

[18] Aliee M, Keeling MJ, Rock KS. Modelling to explore the potential impact of asymptomatic human infections on transmission and dynamics of African sleeping sickness. PLoS Computational Biology. 2021;17(9):e1009367.

[19] Crump RE, Huang CI, Spencer SE, Brown PE, Shampa C, Mwamba Miaka E, et al. Modelling to infer the role of animals in *gambiense* human African trypanosomiasis transmission and elimination in the DRC. PLoS Neglected Tropical Diseases. 2022;16(7):e0010599.

[20] Huang CI, Crump RE, Crowley EH, Hope A, Bessell PR, Shampa C, et al. A modelling assessment of short-and medium-term risks of programme interruptions for *gambiense* human African trypanosomiasis in the DRC. PLoS Neglected Tropical Diseases. 2023;17(4):e0011299.

[21] Antillon M, Huang CI, Sutherland SA, Crump RE, Bessell PR, Shaw AP, et al. Health economic evaluation of strategies to eliminate *gambiense* human African trypanosomiasis in the Mandoul disease focus of Chad. PLoS Neglected Tropical Diseases. 2023;17(7):e0011396.

[22] Kaba D, Koffi M, Kouakou L, N'Gouan EK, Djohan V, Courtin F, et al. Towards the sustainable elimination of human African trypanosomiasis in Côte d'Ivoire using an integrated approach. PLoS Neglected Tropical Diseases. 2023;17(7):e0011514.

[23] Davis CN, Crump RE, Sutherland SA, Spencer SE, Corbella A, Chansy S, et al. Comparison of stochastic and deterministic models for gambiense sleeping sickness at different spatial scales: A health area analysis in the DRC. PLoS Computational Biology. 2024;20(4):e1011993.

[24] Antillon M, Huang CI, Sutherland SA, Crump RE, Brown PE, Bessell PR, et al. Cost-effectiveness of end-game strategies against sleeping sickness across the Democratic Republic of Congo. medRxiv. 2024;p. 2024–12.

[25] NTD Modelling Consortium Onchocerciasis Group. The World Health Organization 2030 goals for onchocerciasis: Insights and perspectives from mathematical modelling: NTD Modelling Consortium Onchocerciasis Group [version 1; peer review: 3 approved]. Gates Open Research. 2019;3:1545.

[26] Crump RE, Huang CI, Knock ES, Spencer SE, Brown PE, Mwamba Miaka E, et al. Quantifying epidemiological drivers of gambiense human African Trypanosomiasis across the Democratic Republic of Congo. PLoS computational biology. 2021;17(1):e1008532.

Annexe A Version française de cet article.

Ceci est la version française de l'article « Measuring elimination of *gambiense* human African trypanosomiasis: A comparison of deceptively different metrics ». Cette traduction a été réalisée à l'aide du site DeepL, et a été éditée et vérifiée manuellement par les auteurs.

Annexe B Résultats supplémentaires

Tous nos résultats ont été générés à partir de huit paramétrages différents. Des figures équivalentes à la Figure 2 sont disponibles en supplément. Le code et les données utilisées pour générer ces figures sont disponibles dans le dépôt de l'Open Science Framework

Annexe C Modèle sous-jacent utilisé pour les simulations

Le modèle utilisé est le même que celui utilisé dans Antillon et al. (2024) [24]. Nous avons utilisé les paramétrages d'ensemble de cet article, en les modifiant légèrement pour supprimer l'amélioration de la surveillance passive en fonction du temps et la fixer plutôt au niveau pré- ou post-amélioration, selon la stratégie simulée. Le paragraphe suivant est une reproduction de la description du modèle tirée de l'article, pour la convenance du lecteur.

Pour cette étude, nous avons utilisé deux variantes du modèle THAg de Warwick publié précédemment [19, 26] consistant en un cadre de modélisation mécaniste et déterministe pour simuler explicitement la transmission entre les humains et éventuellement les animaux via les vecteurs tsé-tsé (voir la section sur la formulation du modèle du supplément d'Antillon et al.). La paramétrisation du modèle a été réalisée individuellement pour chaque variante du modèle (modèles avec et sans transmission animale possible) et a été mise à jour par rapport aux publications précédentes en s'adaptant aux données de l'Atlas de la THA de l'OMS de 2000 à 2020 pour chaque zone de santé de la RDC qui disposait de suffisamment de données - au moins 10 ou 13 points de données pour les modèles sans et avec transmission animale, respectivement (où toute année avec AS et toute année avec une détection passive de cas non zéro comptent comme des points de données individuels). Cela a donné 165 zones de santé avec un ajustement utilisant le modèle sans transmission animale et 156 qui ont été ajustées aux deux variantes du modèle. De plus amples détails sur la procédure d'ajustement statistique sont fournis dans les méthodes supplémentaires. Le modèle « ensemble » est constitué des postérieurs des deux modèles. La proportion d'échantillons provenant des deux modèles a été déterminée par une méthode statistique (facteurs de Bayes) qui mesure la qualité relative de l'ajustement de chaque modèle aux données.
